# Rapid generation of a transgene-free powdery mildew resistant tomato by genome deletion

**DOI:** 10.1038/s41598-017-00578-x

**Published:** 2017-03-28

**Authors:** Vladimir Nekrasov, Congmao Wang, Joe Win, Christa Lanz, Detlef Weigel, Sophien Kamoun

**Affiliations:** 1 The Sainsbury Laboratory, Norwich Research Park, Norwich, NR4 7UH UK; 2 Institute of Digital Agriculture, Zhejiang Academy of Agricultural Sciences, Hangzhou, Zhejiang 310021 P.R. China; 3 Department of Molecular Biology, Max Planck Institute for Developmental Biology, 72076 Tübingen, Germany; 4 Rothamsted Research, Harpenden, Hertfordshire AL5 2JQ UK

## Abstract

Genome editing has emerged as a technology with a potential to revolutionize plant breeding. In this study, we report on generating, in less than ten months, Tomelo, a non-transgenic tomato variety resistant to the powdery mildew fungal pathogen using the CRISPR/Cas9 technology. We used whole-genome sequencing to show that Tomelo does not carry any foreign DNA sequences but only carries a deletion that is indistinguishable from naturally occurring mutations. We also present evidence for CRISPR/Cas9 being a highly precise tool, as we did not detect off-target mutations in Tomelo. Using our pipeline, mutations can be readily introduced into elite or locally adapted tomato varieties in less than a year with relatively minimal effort and investment.

## Introduction

As the world population grows, there is an increasing demand for food. This demand needs to be addressed in a sustainable manner e.g. by creating new crop varieties with valuable traits, such as higher yield, enhanced disease resistance, improved salt and drought tolerance. Traditional plant breeding has been used to generate new crop varieties for decades, but new technologies, such as genome editing, have the potential to generate improved varieties faster and at a lower cost, by precise introduction of favorable alleles into many different, locally adapted elite varieties. Genome editing is achieved using sequence-specific nucleases (SSNs) and results in chromosomal changes, such as nucleotide deletions, insertions or substitutions at specified genetic loci. SSNs include zinc finger nucleases (ZFNs), TAL effector nucleases (TALENs) and, most recently, clustered regularly interspaced short palindromic repeats (CRISPR)/CRISPR-associated protein 9 (Cas9) system^1^. Because certain genetic changes (e.g. nucleotide deletions) introduced using SSNs are indistinguishable from natural mutations, the resulting crop varieties are different from transgenics, aka genetically modified organisms (GMO). In the US, in several cases, genome edited crops have not been regulated, and a decision on regulation of these technologies is forthcoming in Europe^2–4^.

**Figure 1:**
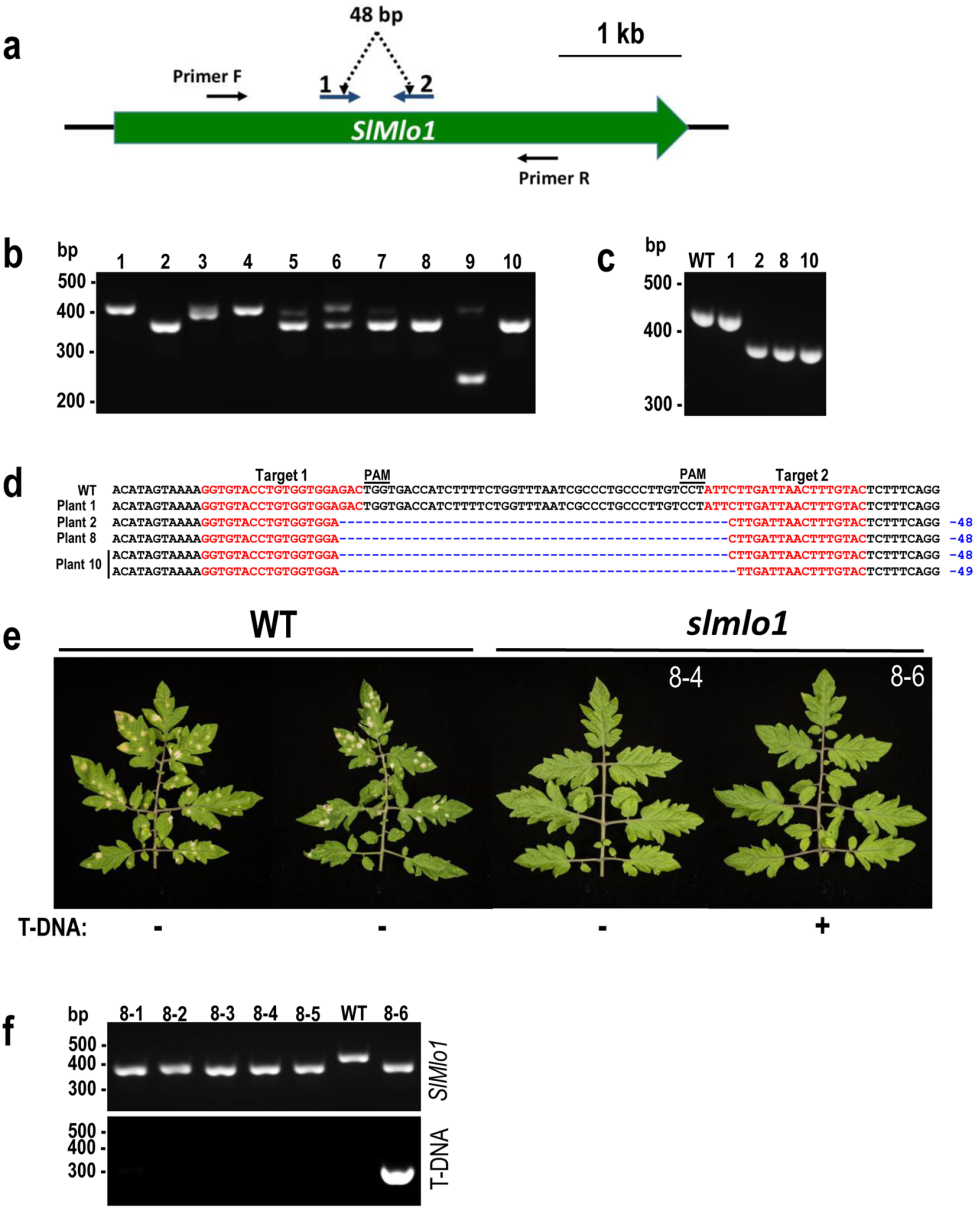
Generating non-transgenic *slmlo1* tomato lines resistant to powdery mildew. (**a**) The *SlMlo1* locus was targeted by two sgRNAs; (**b**) T0 tomato transformants were tested for the presence of deletions using the PCR band shift assay; (**c**) Selected T0 transformants genotyped using the PCR band shift assay alongside wild type (WT); (**d**) *SlMlo1* sequencing reads from selected T0 transformants; (**e**) Leaves of tomato plants inoculated with *Oidium neolycopersici* (5 weeks post inoculation); (**f**) PCR genotyping of the T1 generation for the presence T-DNA and the *slmlo1* mutation. The agarose gels presented in panels (**b** and **c**) were cropped.

In this study we report on generating a tomato variety resistant to the powdery mildew fungal pathogen *Oidium neolycopersici* using the CRISPR/Cas9 technology, which is based on the Cas9 DNA nuclease guided to a specific DNA target by a single guide RNA (sgRNA). Wild-type alleles of *MILDEW RESISTANT LOCUS O* (*Mlo*), which encodes a membrane-associated protein with seven transmembrane domains and is conserved throughout monocots and dicots, confer susceptibility to fungi causing the powdery mildew disease^5^. Therefore, homozygous loss-of-function mutations (*mlo*) result in resistance to powdery mildew. In tomato, there are sixteen *Mlo* genes, *SlMlo1* to *SlMlo16* with *SlMlo1* being the major contributor to powdery mildew susceptibility^6^. Natural non-transgenic loss-of-function *slmlo1* mutants are available in tomato, but introgression of such mutations into an elite variety is a lengthy and laborious process. We aimed at determining the speed and efficiency at which we could generate a transgene-free genetically edited *slmlo1* tomato variety using the CRISPR/Cas9 system. We targeted the *SlMlo1* locus using the double sgRNA strategy as previously described^7^. The two selected targets within *SlMlo1* were positioned on opposite DNA strands and spaced 42 bp apart (Fig. 1a). Ten primary transformants (T0) carrying the T-DNA expressing Cas9 and sgRNAs were analysed for altered electrophoretic mobility of PCR amplicons as an indication of modifications in *SlMlo1*. Eight out of ten tested T0 transformants showed a mobility shift indicating the presence of mutations (Fig. 1b). We selected three transformants (2, 8 and 10) that showed a band shift consistent with the expected deletion of 48 bp when Cas9 cuts 3 bp away from the PAM motif within the target sequence^8^ (Fig. 1c). The PCR amplicons were cloned and individual clones sequenced (Fig. 1d). All three transformants that showed the expected band shift proved to carry homozygous (transformants 2 and 8) or biallelic (transformant 10) mutations at the *SlMlo1* locus.

To generate non-transgenic *slmlo1* tomato lines, we segregated away the T-DNA by selfing T0 transformant 8 and cultivating next generation (T1) plants. In the progeny, we identified five *slmlo1* T-DNA-free individuals (Fig. 1f). Disease resistance assays with the powdery mildew fungus *Oidium neolycopersici* revealed that all the *slmlo1* mutant plants were fully resistant to the pathogen, whereas all tested wild-type plants were susceptible (Fig. 1e). The *slmlo1* mutant plants were not only morphologically similar to the wild type (Supplementary Fig. 1), but they also produced harvested fruit weight similar to the wild type (Supplementary Fig. 2).

**Figure 2:**
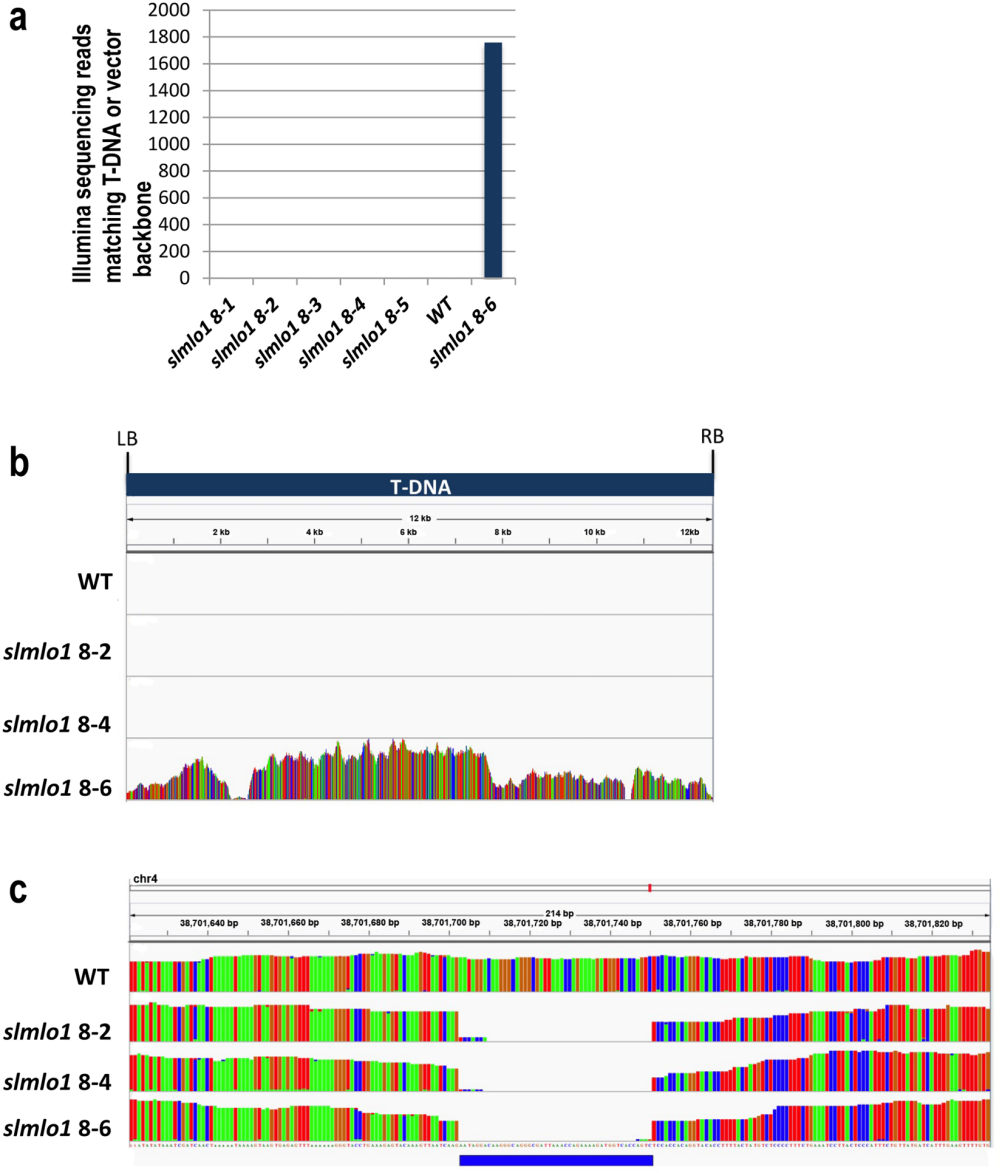
Illumina sequencing data. (**a**) Quantification of Illumina sequencing reads matching the T-DNA or vector backbone in wild type and *slmlo1* T1 progeny lines; (**b**) Coverage of the T-DNA by Illumina reads; (**c**) Coverage of the *SlMlo1* locus by Illumina reads.

To verify that the *slmlo1* mutants are indeed free from any T-DNA or transformation vector sequences, and to confirm the presence of a homozygous deletion in *SlMlo1*, we Illumina sequenced genomic DNA from the five T1 T-DNA free *slmlo1* segregants (8-1, 8-2, 8-3, 8-4 and 8-5), one *slmlo1* T1 segregant carrying the T-DNA (8-6), as well as the wild type to at least 28x genome coverage (Supplementary Table 1). We first mapped the reads to the complete vector used for T-DNA transformation, allowing for 4% mismatches. In the *slmlo1* 8-6 plant, in which we had confirmed the presence of T-DNA sequences by PCR, 1,757 reads matching the T-DNA or transformation vector backbone were detected. In contrast, no reads matching the T-DNA or vector were detected in mutants *slmlo1* 8-2 and 8-4, while only 1 or 2 reads were detected in lines 8-1, 8-3, and 8-5, as well as the wild type (Fig. 2a,b). These reads are most likely the result of low levels of carry-over contamination between runs due to the extreme sensitivity of the DNA sequencing method. We conclude that mutant lines *slmlo1* 8-2 and 8-4 are unambiguously free of transgenic sequences. The mutants were confirmed to carry the *slmlo1* 48 bp deletion in a homozygous configuration and to be clearly different from the parental line in this locus based on the whole genome shotgun sequence analysis (Fig. 2c).

We also evaluated the extent to which the genomes of *slmlo1* mutants suffered from off-target mutations caused by the CRISPR/Cas9 system. We generated a list of 145 potential off-targets for both CRISPR/Cas targets with up to 4 mismatches using the Cas-OFFinder online tool (http://www.rgenome.net/cas-offinder/) (Supplementary Table 2). We performed the off-target analysis by mapping the Illumina reads from wild-type and *slmlo1* 8-2 genomes to the tomato reference genome. Out of 145 putative off-targets, 140 were covered by Illumina reads in both the wild type and *slmlo1* 8-2 mutant (Supplementary Table 2). The results of the DNA base calling analyses are presented in Supplementary Tables 3 and 4. Side-by-side comparison of DNA bases called in the wild type and *slmlo1* 8-2 revealed that 12 out of 39 differences (31%) were confined to the targeted *SlMlo1* locus on chromosome 4 (Supplementary Tables 5 and 6). The remaining 27 out of 39 differences (69%) that we observed in genomic regions outside of the targeted *SlMlo1* locus did not indicate off-target effects (Supplementary Tables 5 and 6). For example, a region of several Mb on chromosome 9 was poorly covered by reads in both the wild type and *slmlo1* 8-2, with several potential off-targets lacking reads from both the wild type and *slmlo1* 8-2, but one off-target, at position 38,542,554, was covered by just one read in wild type and no reads in *slmlo1* 8-2 (Supplementary Table 2 and Supplementary Fig. 3). We note that this sequence carries four mismatches relative to target 2, with two of the mismatches close to the PAM motif, which are expected to dramatically decrease the ability of CRISPR/Cas9 to recognise it^8^. Therefore, it is likely to be by chance that the off-target was covered in wild type but not in the *slmlo1* 8-2 line. We also detected heterozygous SNPs at two positions in the *slmlo1* 8-2 mutant (chr8, positions 21,452,901 and 21,452,908), where the wild type appeared to be homozygous (Supplementary Tables 5 and 6). In addition, we detected heterozygous SNPs at two positions in wild type (chr1, position 43,886,749; chr2, position 2,894,674), where the *slmlo1* 8-2 mutant appears to be homozygous (Supplementary Tables 5 and 6). Visual examination of the sequence reads at these sites revealed that homozygous SNP calls at all four positions were an artifact resulting from the 0.25 threshold for calling heterozygous positions with both wild type and *slmlo1* 8-2 showing heterozygous calls when the threshold was lowered to 0.16 (Supplementary Table 7, and Supplementary Figs 4 and 5). Since our inbred wild-type strain is more likely to be homozygous, these heterozygous calls likely resulted from mis-mapping of sequences related to the target locus that are present in the wild type GCR758 accession^9^, a derivative of the Moneymaker cultivar, and its descendants, but not in the Heinz 1706 strain from which the reference genome sequence was assembled. In conclusion, we did not find credible evidence for mutations at potential off-target positions within the genome of the *slmlo1* 8-2 line. Our results suggest that CRISPR/Cas9 can be a highly precise genome editing tool when applied in tomato consistent with other recent reports in plants^10, 11^.

We named the powdery mildew resistant *slmlo1* tomato variety ‘Tomelo’. We have unambiguously demonstrated by whole-genome sequencing that Tomelo is non-transgenic, i.e. it does not carry any foreign DNA sequences. Importantly, the 48 bp deletion at *SlMlo1* in Tomelo is indistinguishable from naturally occurring mutations. We generated Tomelo in only 9.5 months from the DNA transformation step to recovery of second generation transgene-free segregants (Supplementary Fig. 6). Therefore, using our pipeline, the *slmlo1* mutation could be readily introduced into elite or locally adapted varieties in less than a year with relatively little effort or investment. Although the status of genetically edited crop varieties like Tomelo is still debated by regulatory authorities throughout the world, some countries, such as the US, consider transgene-free genetically edited crops as non-GMO^2, 3^. We hope that plant varieties, such as Tomelo, are adopted world-wide with similar regulatory burden as traditionally bred varieties to meet the food demands of the growing world’s population and promote competitiveness of the agrobiotech sector by reducing chemical input.

## Methods

### Plasmid construction

The plasmid pAGM4723::Cas9_sgRNA1_sgRNA2 (Supplementary sequence file 1) was assembled as previously described^7^. Primers Mlo1_target1_f and sgRNAr were used to amplify sgRNA1, and primers Mlo1_target1_f and sgRNAr were used to amplify sgRNA2 (Supplementary Table 8).

### Plant transformation

GCR758^9^, a derivative of tomato cultivar Moneymaker, was transformed with the pAGM4723::Cas9_sgRNA1_sgRNA2 construct as previously described^12^. T0 transgenic plants were selected in the medium with kanamycin and then transferred into soil.

### Plant DNA extraction

The DNA was extracted from the plant tissue using 500 ul of CTAB buffer (2% CTAB, 1.42 M NaCl, 20 mM EDTA and 100 mM Tris pH 8) for 30 min at 60 °C. Then 300 ul of chlorophorm was added. The mix was vortexed and then spun for 20 min at 4,500 rpm. The genomic DNA was precipitated from the supernatant with 0.7 volumes of isopropanol at −20 °C overnight. The mix was centrifuged for 15 min at 12,000 rpm and the pellet was then washed with 1 ml of ice cold 70% ethanol. The pellet was dried and resuspended in water.

### Detection of Cas9-induced deletions and T-DNA in plant genomic DNA using PCR genotyping

The *SlMlo1* locus (**Solyc04g049090**) was PCR amplified using Phusion DNA polymerase (New England Biolabs) across targets 1 and 2 using SlMloseq3f and SlMloseq1r primers. The PCR products were run on a 3% agarose gel, extracted from the gel using the Qiagen gel extraction kit and cloned into pCR-Blunt II-TOPO vector. Eight individual clones were sequenced using M13F and M13R primers. The T-DNA was detected by PCR amplification using primers Cas9_2f and Cas9_2r.

### Whole genome Illumina sequencing

To avoid amplification artifacts introduced during library preparation for Illumina sequencing all libraries were prepared following the TruSeq DNA PCR-free library preparation guide (Cat. No: FC-121-9006DOC) and using TruSeq DNA PCR-Free LT kit Set A (Cat. No: FC-121-3001). A starting amount of 1 μg DNA was sheared to an average insert size 350 bp by AFA (S2, Covaris) followed by end repair, A-tailing and adapter ligation. We obtained sufficient paired-end DNA for validation and sequencing. The quality of the libraries was checked on a Bioanalyzer (Agilent Technologies) using a High Sensitivity DNA chip and 1 µl of diluted DNA library. Quantification was done by Qubit (Invitrogen) and qPCR.

DNA Sequencing was performed on HiSEQ3000 sequencing system (Illumina), equipped with on-instrument HiSeq control software (HCS) version 3.3.20 and Real time analysis (RTA) version2.5.2. Cluster generation was performed on a cBot (recipe: HiSeq_3000_4000_HB_Exclusion_Amp_v1.0, Illumina) using a patterned flow cell and reagents from PE Cluster Kit (Cat. No: PE-410-1001, Illumina) according to the manufacturer’s instructions. The libraries were sequenced in 2 lanes paired end with 151 bp read length and 8 bp index read using 300cycle HiSEQ3000 SBS kits (Cat No: FC-410-1003). The library was diluted with Resuspension Buffer (Illumina) to 2 nM before clustering. The final DNA concentration on the flowcell was 200 pM which resulted in 2 × 329 M reads (past filter) in the first lane and 2 × 319 M reads in the second lane.

### Off-target mutation analysis

Each tomato line was analyzed separately with the SHORE pipeline^13^, using BWA aln (v0.6.2)^14^ with option “-n 0.05” to map the reads to the *Solanum lycopersicum* (version SL2.40.26; ftp://ftp.ensemblgenomes.org/pub/release-26/plants/fasta/solanum_lycopersicum/dna/) reference genome. As a result, .bam files for *slmlo1* 8-2 and WT have been generated.

We produced a list of 145 potential off-targets for both CRISPR/Cas targets with up to 4 mismatches using the Cas-OFFinder online tool (http://www.rgenome.net/cas-offinder/) (Supplementary Table 2). For each predicted off-target, we calculated nucleotide frequencies based on the coverage of all 23 nucleotide positions (Supplementary Tables 3 and 4). A SNP was called if the rate of a differential nucleotide, as compared to the reference genome, equaled 0.25 or more (Supplementary Table 5).

### Pathotest with Oidium neolycopersici

The tomato lines were infected with airborne spores of *Oidium neolycopersici* propagated on wild type GCR758 tomato plants in an isolated growth room. The experiment was done twice with similar results.

### Analysis of the fruit harvest from *slmlo1* vs wild type tomato plants

The fruits were collected on nine different days and plants that produced fruits on more than one day were included into the analysis. The statistics was done using the algorithm described^15^.

## Electronic supplementary material

Supplementary Information

Supplementary Information

Supplementary Information

Supplementary Information

Supplementary Information

Supplementary Information

Supplementary Information
