# Supplementary material for: Rapid generation of a transgene-free powdery mildew resistant tomato by genome deletion
Source: Sci Rep. 2017 Mar 28;7:482. doi: 10.1038/s41598-017-00578-x (PMC5428673; doi:10.1038/s41598-017-00578-x)
Supplement: Supplementary file 1 — Supplementary Information [file 41598_2017_578_MOESM1_ESM.pdf]

SUPPLEMENTARY INFORMATION

Rapid generation of a transgene-free powdery mildew resistant tomato by genome deletion

Vladimir Nekrasov[1§], Congmao Wang[2], Joe Win[1], Christa Lanz[3], Detlef Weigel[3*] and Sophien Kamoun[1*]

[1]The Sainsbury Laboratory, Norwich Research Park, Norwich NR4 7UH, UK
[2]Institute of Digital Agriculture, Zhejiang Academy of Agricultural Sciences, Hangzhou, Zhejiang, 310021, PR China
[3]Department of Molecular Biology, Max Planck Institute for Developmental Biology, 72076 Tübingen, Germany

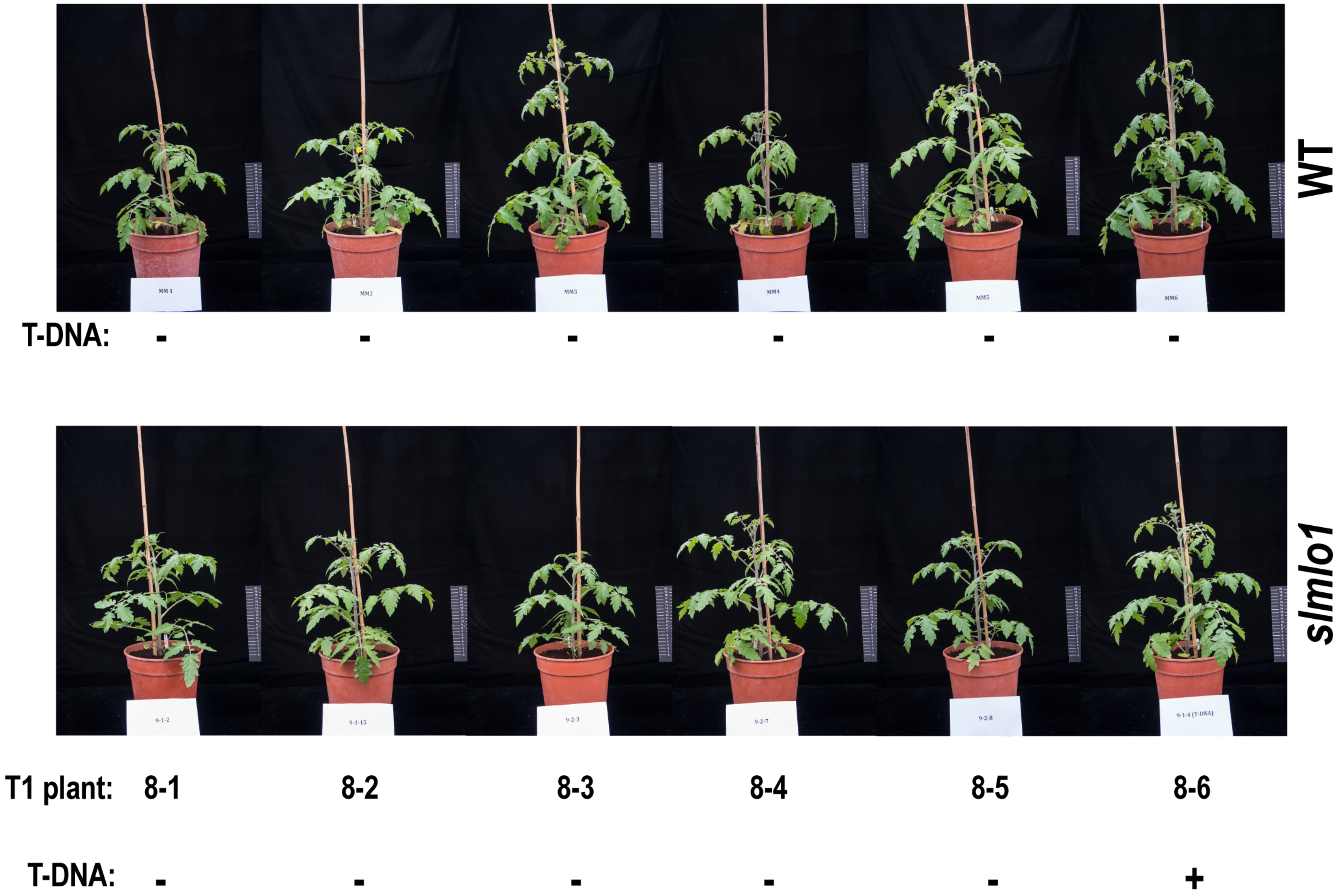

Supplementary Figure 1.

Wild type and *slmlo1* tomato plants at the 8 week stage.

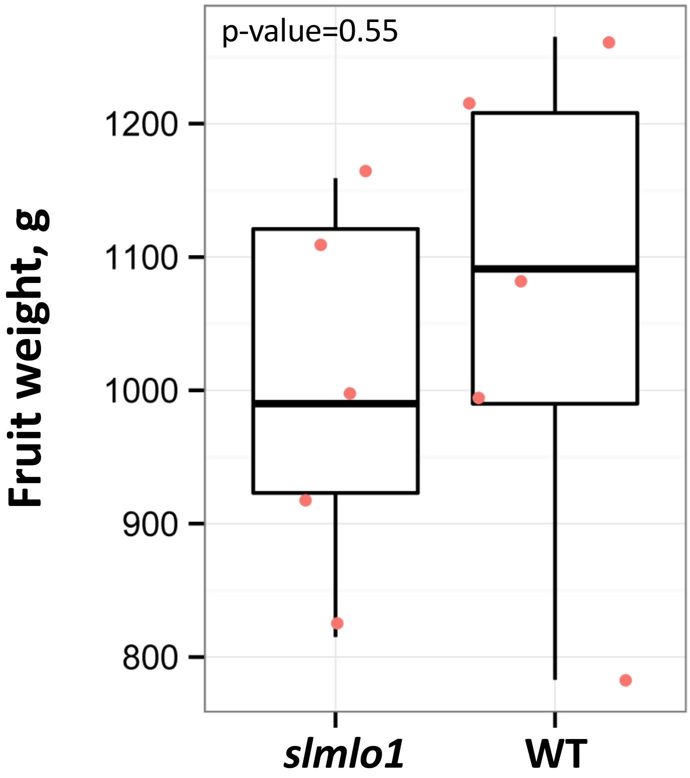

Supplementary Figure 2.
Fruit harvest from wild type (WT) and *slmlo1* tomato plants. The graph represents average fruit harvest from five wild type and five *slmlo1* plants.

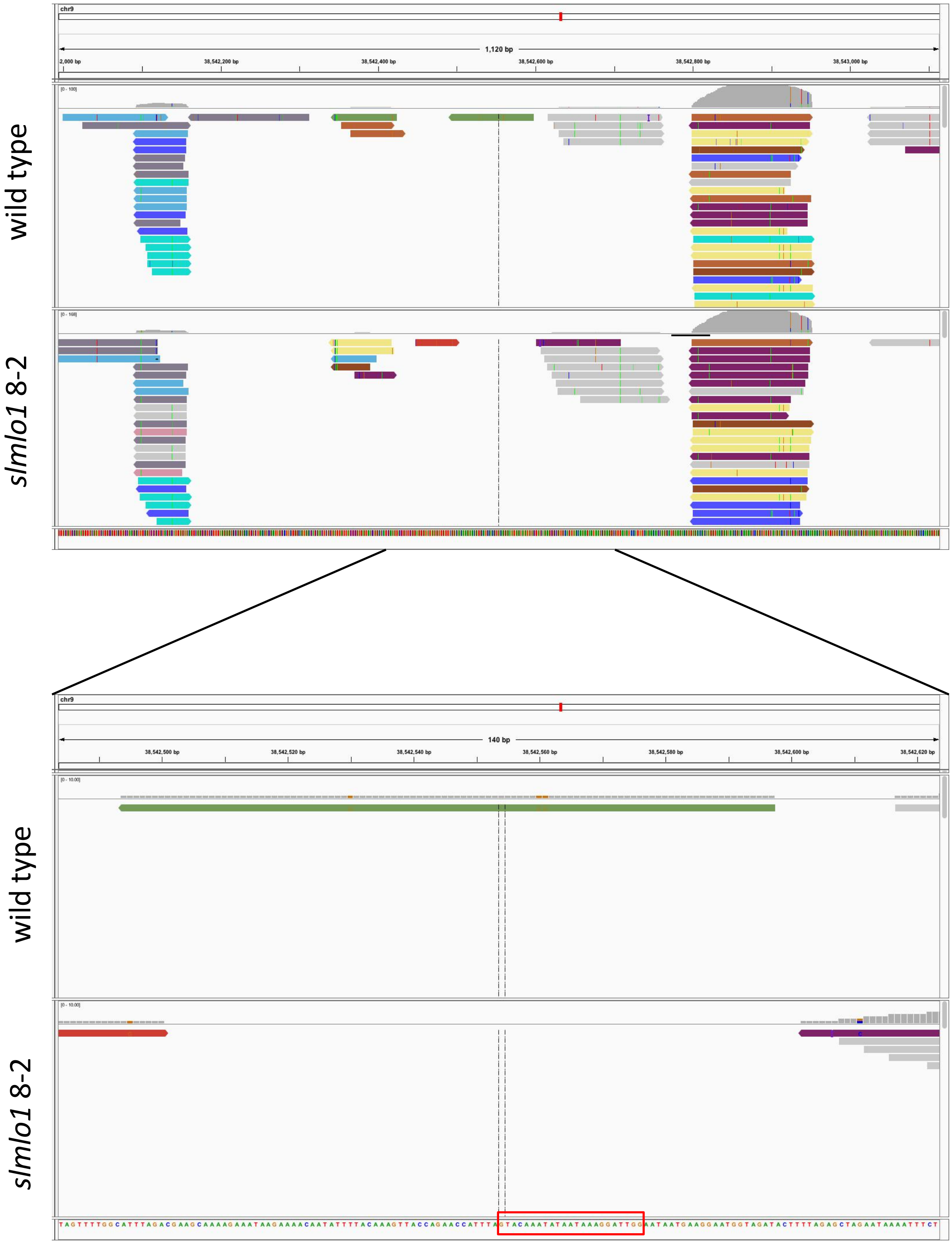

Supplementary Figure 3.
Illumina coverage of the off-target on chromosome 9, position 38,542,554.

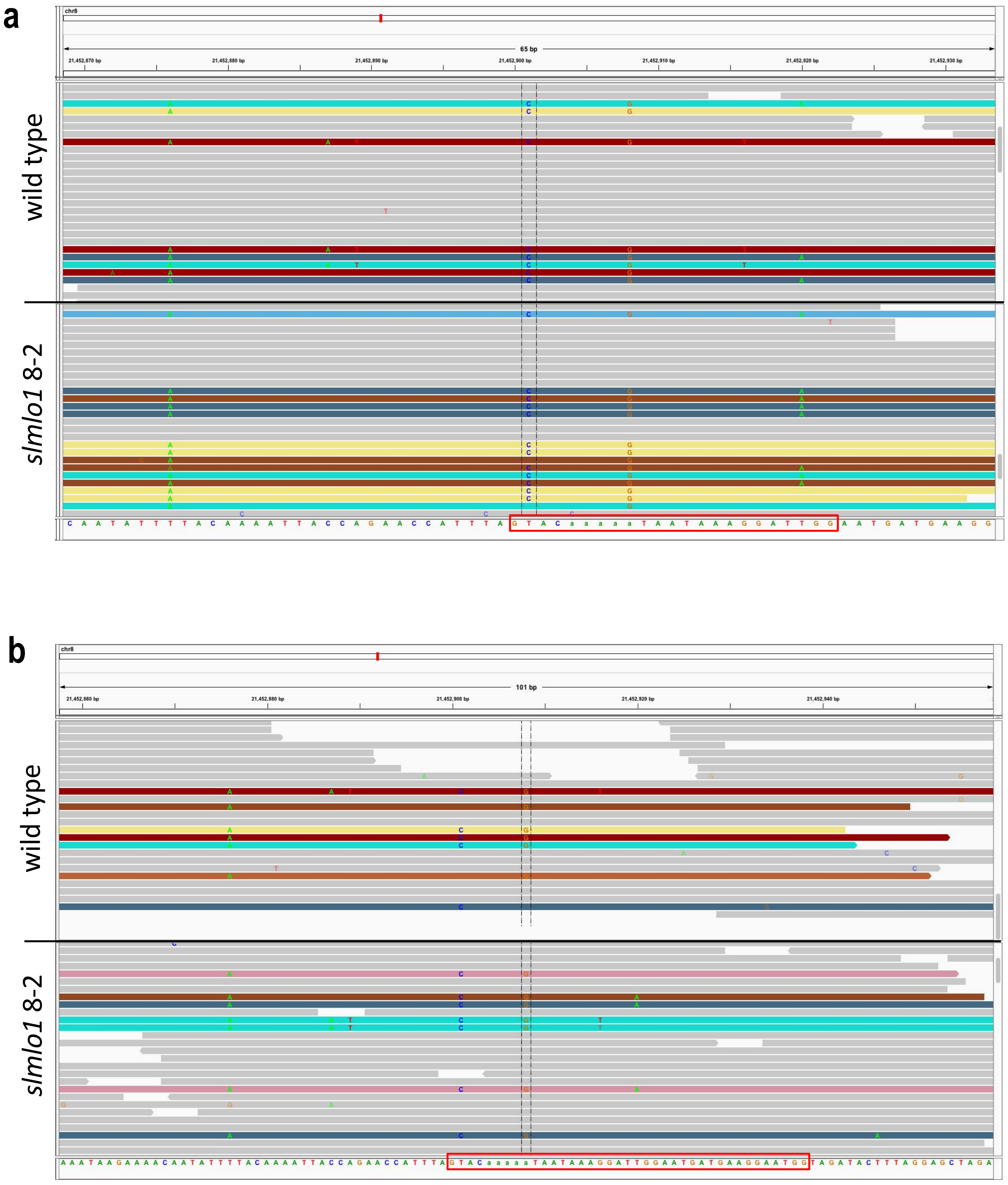

Supplementary Figure 4.
Illumina data showing heterozygous SNPs on chromosome 8 at positions 21,452,901 (a) and 21,452,908 (b) in both wild type and *slmlo1* 8-2.

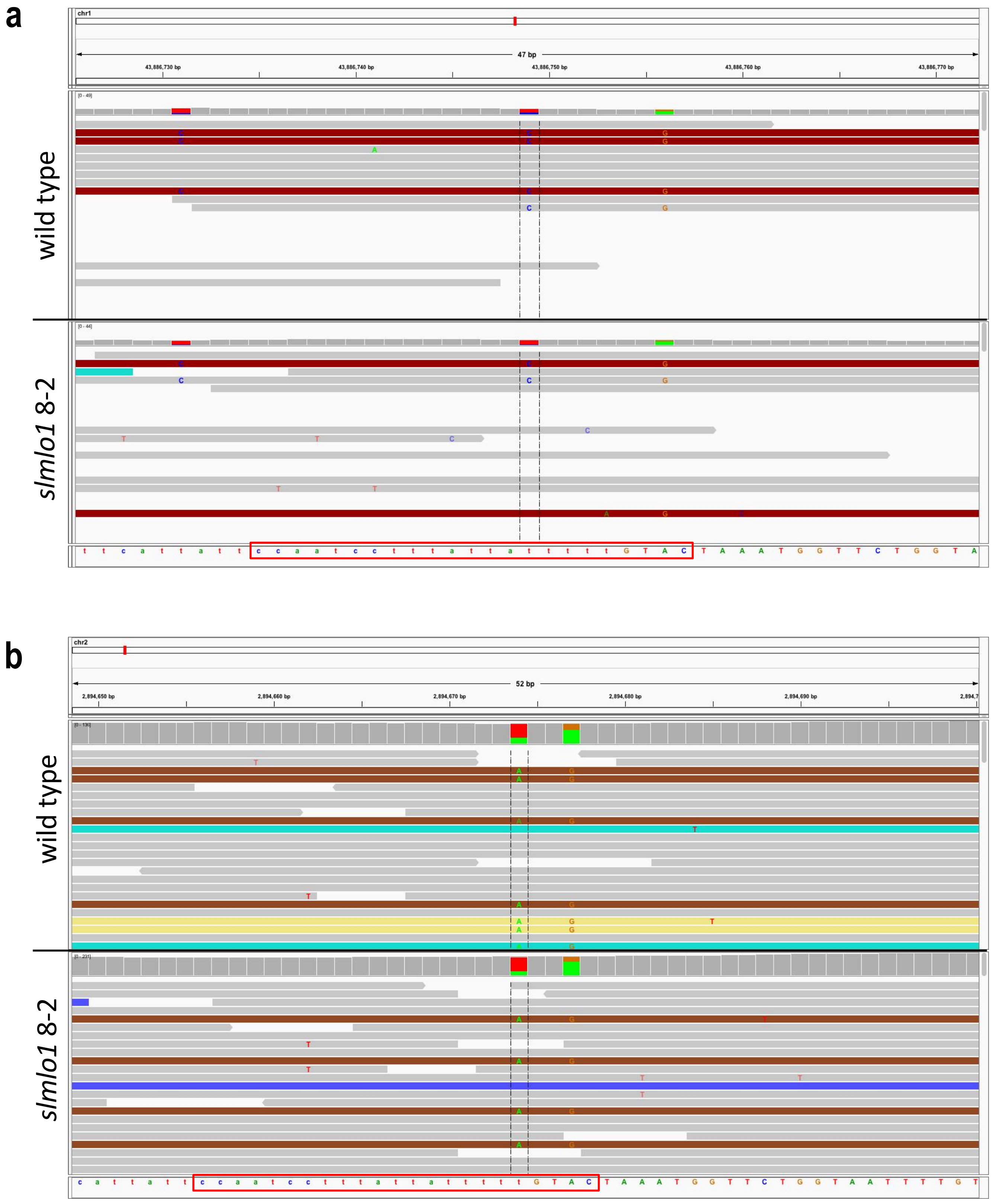

Supplementary Figure 5

Illumina data showing heterozygous SNPs on chromosome 1 at position 43,886,749 (a) and chromosome 2 at position 2,894,674 (b) in both wild type and *slmlo1* 8-2.

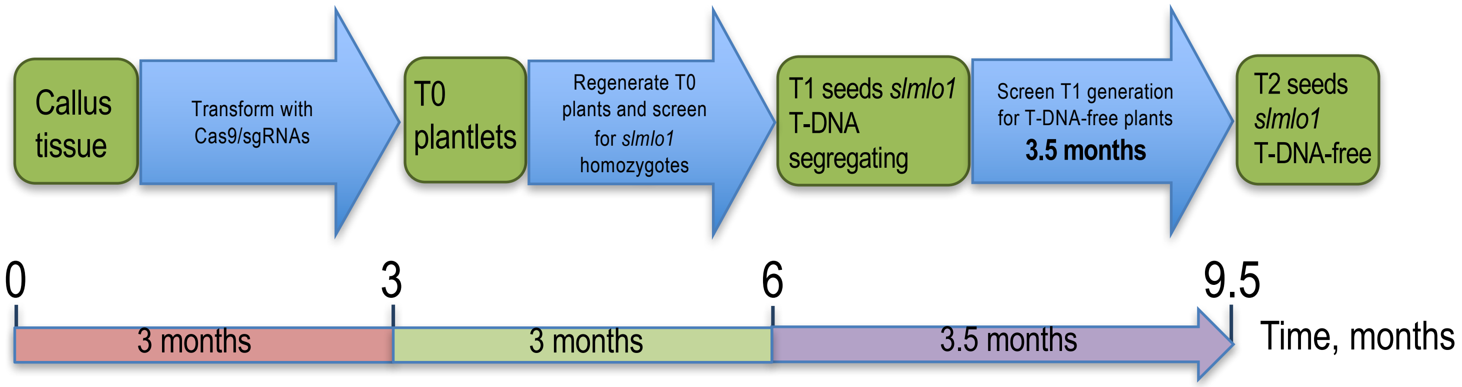

Supplementary Figure 6.
The timescale of generating nontransgenic *slmlo1* knockout tomato lines.

Supplementary Table 1. Summary of the Illumina sequencing data.

	wild type	*slmlo1*					
		**8-1**	**8-2**	**8-3**	**8-4**	**8-5**	**8-6**
**Total Sequences (paired reads)**	335,281, 574	102,736, 318	447,224,1 42	84,559, 994	89,901, 369	95,910, 142	86,911, 928
**Sequences flagged as poor quality**	0	0	0	0	0	0	0
**Sequence length**	35-151	35-151	35-151	35-151	35-151	35-151	35-151
**%GC**	35	35	35	35	35	35	34
**Genome coverage**	112X	34X	149X	28X	30X	32X	29X

Supplementary Table 8. Primers used in this study.

Primer name	Primer sequence
Mlo1_target1_f	TGTGGTCTCAATTGGTGTACCTGTGGTGGAGACGTTTTAGAGCTAGAAATAGCAAG
Mlo1_target2_f	TGTGGTCTCAATTGTACAAAGTTAATCAAGAATGTTTTAGAGCTAGAAATAGCAAG
sgRNAr	TGTGGTCTCAAGCGTAATGCCAACTTTGTAC
Cas9_2f	GAGGAAGACAAGACTATCACTCCCTGGAACTTCGAG
Cas9_2r	GAGGAAGACAAATCTTCTTTGAGCTGTTTCACGGTAACT
SlMloseq3f	AAGTATGTTCATGATTGACTAAAG
SlMloseq1r	CCAAGTTAGGAGTTTGTACCG
M13F	GTTGTAAAACGACGGCCAGT
M13R	CAGGAAACAGCTATGACC
